# Statistical Assessment of Discrimination Capabilities of a Fractional Calculus Based Image Watermarking System for Gaussian Watermarks

**DOI:** 10.3390/e23020255

**Published:** 2021-02-23

**Authors:** Mario Gonzalez-Lee, Hector Vazquez-Leal, Luis J. Morales-Mendoza, Mariko Nakano-Miyatake, Hector Perez-Meana, Juan R. Laguna-Camacho

**Affiliations:** 1 Facultad de Ingeniería en Electrónica y Comunicaciones, Universidad Veracruzana, Av. Venustiano Carranza S/N, Poza Rica Veracruz C.P. 93390, Mexico; javmorales@uv.mx; 2 Facultad de Instrumentación Electrónica, Universidad Veracruzana Lomas del Estadio S/N, Xalapa Veracruz C.P. 91090, Mexico; hvazquez@uv.mx; 3 Consejo Veracruzano de Investigación Científica y Desarrollo Tecnológico (COVEICYDET), Av. Rafael Murillo Vidal No. 1735, Cuauhtemoc, Xalapa Veracruz C.P. 91069, Mexico; 4 Seccion de Estudios de Posgrado e Investigacion, Instituto Politécnico Nacional, Av. Santa Ana No. 1000, Del. Coyacan, Ciudad de Mexico C.P. 04440, Mexico; mnakano@ipn.mx (M.N.-M.); hmperezm@ipn.mx (H.P.-M.); 5 Facultad de Ingeniería en Mecánica Electrica, Universidad Veracruzana, Av. Venustiano Carranza S/N, Poza Rica Veracruz C.P. 93390, Mexico; jlaguna@uv.mx

**Keywords:** fractional calculus, Gaussian watermarks, statistical assessment, false positive rate, semi-fragile watermarking system

## Abstract

In this paper, we explore the advantages of a fractional calculus based watermarking system for detecting Gaussian watermarks. To reach this goal, we selected a typical watermarking scheme and replaced the detection equation set by another set of equations derived from fractional calculus principles; then, we carried out a statistical assessment of the performance of both schemes by analyzing the Receiver Operating Characteristic (ROC) curve and the False Positive Percentage (FPP) when they are used to detect Gaussian watermarks. The results show that the ROC of a fractional equation based scheme has 48.3% more Area Under the Curve (AUC) and a False Positives Percentage median of 0.2% whilst the selected typical watermarking scheme has 3%. In addition, the experimental results suggest that the target applications of fractional schemes for detecting Gaussian watermarks are as a semi-fragile image watermarking systems robust to Gaussian noise.

## 1. Introduction

Digital watermarking has gained popularity in the past few decades as a copyright enforcement tool. It is an active research field that includes applications such as data authentication and data indexing among other practical applications [1,2,3]. The scenario of copyright enforcement is as follows: the copyright holder wants to exploit some digital media, so he embeds a watermark under the premise that, in case of an unauthorized person exploiting the media, the copyright holder would be able to demonstrate in court that his watermark was embedded in the media and hence he owns all rights to the media.

A watermarking system embeds a signal, called the watermark, into another signal known as the cover; a cover might be digital media such as an image, audio, video, or other digital media. Most of the proposed watermarking systems generate a pseudo-random signal (the watermark) using a user’s key and then embeds this watermark into the cover; conversely, the watermarking system is able to detect the watermark or even retrieve it from the watermarked cover. If watermark samples are in the set {−1,1}, then the watermark is called binary; sometimes, designers let the watermark be a pseudo-random sequence with Gaussian distribution, this kind or watermark is called Gaussian Watermark.

A watermarking system can have two types of errors during its attempt to detect a watermark:

**Error** **type I:** The system failed to find a watermark; this is called False Negative (FN).
**Error** **type II:** The system found a given watermark even when either no watermark or another watermark was embedded; this is called False Positive (FP).

A FP is considered flawed that must be avoided because this might lead to a legal dispute on the copyrights of the digital media. For this reason, systems that exhibit a high FPP are impractical and thus excluded from literature. Usually, a watermarking system has a negligible FPP for detecting binary watermarks; conversely, some systems might have a high FPP when detecting Gaussian watermarks.

**Figure 1:**
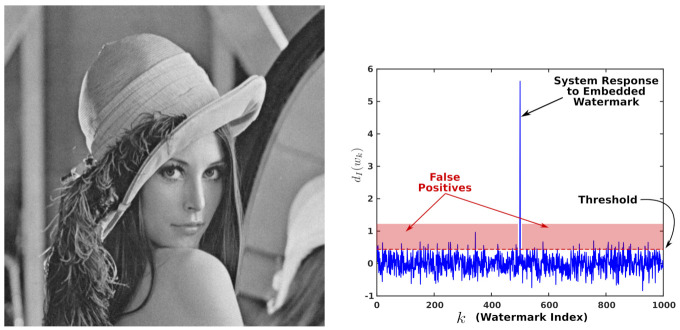
Faulty detection of a Gaussian watermark due to False Positives. (**left**) Watermarked image. g=5, PSNR=34.14 dB. (**right**) The systems verify the presence of several watermarks; the cases that fall in the red zone are False Positives.

To clarify this issue, consider the following example: Figure 1 (left) shows an image watermarked with a Gaussian watermark. Only one watermark was embedded; however, the system detects several watermarks as if they were actually embedded as shown in Figure 1 (right). Assuming that a court acknowledges as the copyright owner any individual who claims the rights to some digital media granted, he can prove that the watermarking system detects his watermark within the media. Under these conditions, an attacker would have to search for a watermark that produces a positive detection and could then claim ownership of the media, causing a legal dispute.

To help to mitigate this issue, we proposed in a previous paper to replace the detection equations of watermarking systems to reduce the FPP. Although results were interesting and seem promising, our tests were not conclusive due to the low number of images in the database used in the experiments; thus, the purpose of this paper is to fill the remaining gaps in our previous proposal by analyzing the cases we left unexplored using a bigger image database. In this paper, we put our early proposal on a firmer basis, we:

- Assessed statistically meaningful results by extending the data set up to 10,000 images.
- Carried out a statistical analysis to compare the FPP of the original watermarking scheme versus the corresponding version with detection equations derived from fractional calculus.
- Evaluated the quality of both schemes as a watermark detector by comparing their ROC curves.
- Examined the successful detection rate after performing a number of signal processing operations on the watermarked images to define robustness of the system and recommend target applications of fractional detector equations.
- Complemented our previous study about binary watermarks with this study about Gaussian watermarks.

With this results as a basis, we expect designers of watermarking systems to take advantage of Gaussian watermarks when appropriate to meet his design goals. At the moment, it is difficult to detect watermarks using simple equations, so we look forward to provide an alternative to reuse previously proposed schemes by using fractional calculus based equations.

The usage scenarios for such schemes include:

- The system designer wants to enhance the discriminative power of a system already proposed.
- The watermark is some information that closely holds the Gaussian distribution.
- The complexity of the watermarking system has to be low.

Another scenario will be discussed later.

The rest of the paper is organized as follows: in Section 2, we review the background of the analyzed watermarking scheme. A discussion about related works is presented in Section 3. Section 4 presents a Fractional Scheme for watermarking. In Section 5, we discuss the materials and methods of analysis used to carry out the experiments; next, in Section 6, we present the experimental results; then, in Section 7, we discuss the experimental results and present the conclusions, and finally the references in the last section.

## 2. The Watermarking Model

Before continuing with the background fundamentals, let us define the terminology used in the remainder of this paper. One often refers to different watermarks, so we will call the set of different watermarks W; $w_{k}$ is the k-th watermark of the set W and $w_{k}\left[i\right]$ denotes the i-th sample of the k-th watermark. The set of images that serve as covers is X; similarly, $x_{k}$ denotes the k-th image and $x_{k}\left[i\right]$ is the i-th sample of the k-th. $y_{k}\left[i\right]$ is the i-th sample of the k-th watermarked image. Note that, although we are focusing on images, we will use one index for the sake of simplicity, so consider i=(r,c) a coordinate pair of the image.

**Figure 2:**
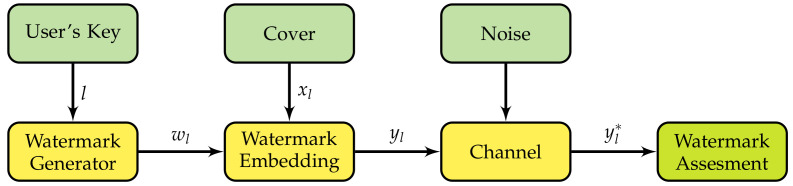
General model of watermarking as a communication process.

A simple model approach to watermarking is to make analogies to the field of the theory of communications. In this context, we assume that the watermark is transmitted through a communications channel as pictured in Figure 2. The model has the following variables: the cover which is a signal used as host for the watermark; a user’s key as input for a pseudo-random number generator, and the embedding gain which is related to the watermark’s energy. In an ideal scenario, the cover does not distort the watermark; however, in practice, this can not be achieved, so the effects of the cover on a watermark are modeled as the distortion caused by the channel. Attacks to the watermark are modeled as noise. An attack is a signal processing operation performed on the watermarked with the goal of making the watermark undetectable by the watermarking system.

### 2.1. Watermark Embedding

There are two basic rules for embedding watermarks: the additive rule and the multiplicative rule. We will focus on the additive embedding rule since it is widely used in most related works.

**Figure 3:**
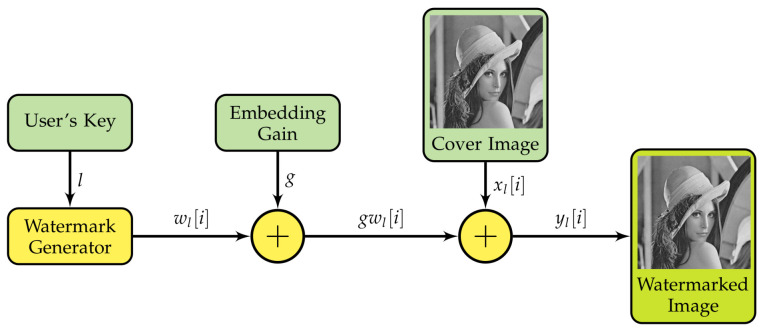
Watermark embedding scheme.

The watermarking system embeds the watermark $w_{l}$ into the cover $x_{l}$ producing the watermarked signal $y_{l}$ as shown in Figure 3. This scheme uses the additive rule defined as:

(1) $$y_{l}=x_{l}+gw_{l},$$

where $y_{l}$ is the watermarked signal and *g* is the embedding gain.

### 2.2. Watermark Detection

**Figure 4:**
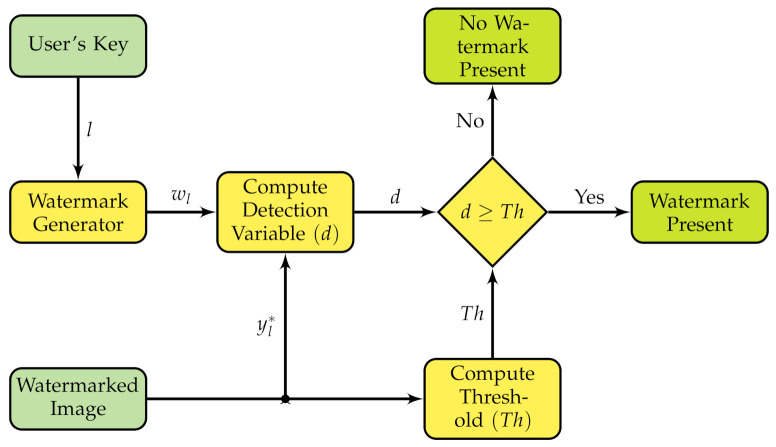
Block diagram of the watermark detection process.

A typical watermark system assesses the presence of the watermark by computing two statistics: a decision variable which is a measurement of the presence of the watermark within the watermarked image, and a threshold that helps to decide if the watermark is present or absent. If the decision variable is greater than or equal to the threshold, then the watermark was detected; otherwise, the watermark is absent as shown in Figure 4.

Most watermarking systems have detected watermarks using the cross-correlation formula since the early works on watermarking; an example is the highly influential paper by Cox et al. [3]. The watermarking system uses the received and possible noisy watermarked media ($y_{l}^{*}$) for detecting the watermark; first, it computes a decision variable $d_{I}\left(w_{l}\right)$ as follows:

(2) $$d_{I}\left(w_{l}\right)=\frac{1}{N}\sum_{i=1}^{N}w_{l}\left[i\right]y_{l}^{*}\left[i\right];$$

Next, the system compares $d_{I}\left(w_{l}\right)$ to a threshold ($Th_{I}\left(w_{l}\right)$) and, if $d_{I}\left(w_{l}\right)\geq Th_{I}\left(w_{l}\right)$, then the detection is positive; the threshold is computed using the following equation [4]:

(3) $$Th_{I}\left(w_{l}\right)=3.3\sqrt{2\frac{\sigma^{2}}{N}}$$

where $\sigma^{2}$ is the variance of $y_{l}^{*}$.

**Figure 5:**
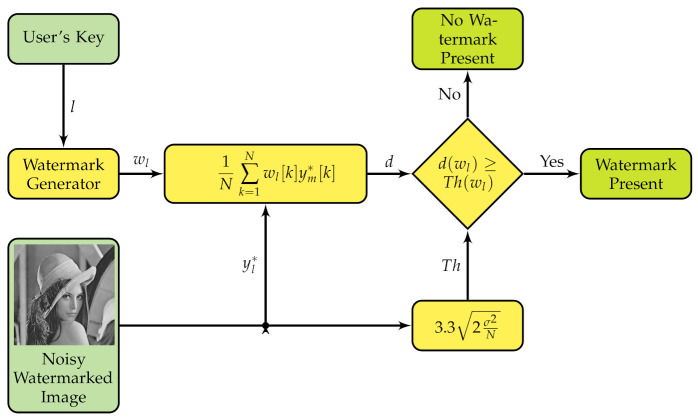
Integer watermark detecting scheme.

Many state-of-the-art algorithms use (1)–(3) for inserting and detecting the watermark as discussed later in this paper. We will call (2) and (3), the integer equation set—hence, the subscript *I* of the detection variable set. A watermarking scheme based on Equations (1)–(3) is shown in Figure 5.

The decision of the system for the integer equation set is computed as:

(4) $$D_{I}\left(w_{l}\right)=\left\{\begin{array}{cc} 1 & d_{I}\left(w_{l}\right)\geq Th_{I}\left(w_{l}\right)\\ 0 & \mathrm{Otherwise} \end{array}\right.,$$

Many works use (1) and (2) to embed and detect watermarks respectively as discussed in next section.

## 3. Works Related to Watermarking Based on Fractional Calculus

On the other hand, Fractional Calculus (FC) has gained attention in recent years; for example, Refs. [5,6,7,8] are good references that cover the basics on FC ranging from introductory to advanced FC theory. Many scientists used it for modeling several physical phenomena with applications to engineering; for example, in [9,10,11], the authors present applications of FC to the analysis of control systems. In [12,13,14], the authors present applications to Digital Filters design. In [15,16], the authors discuss an approach to linear systems analysis for both continuous and discrete cases. Researchers already started to develop FC applications to watermarking; related works exhibit a tendency to adapt (1) and (2) for working with fractional calculus based approaches.

Some authors use a fractional derivative for watermarking since there is a relationship between the order of the derivative and the resulting function; this relationship is difficult to establish. For example, the authors of [17] use the Grünwald–Letnikov fractional operator for computing a pseudo-random sine function, allowing two fractional orders α and β to act as keys. The authors claim that this scheme is robust toward occlusion attack; however, this is the only test they reported. The work [18] is similar to [17]. The main difference between those works is that authors of [18] use the fractional Cauchy formula for the sine function. Authors report that the system is robust; nevertheless, their results are supported by the test in just one image lacking evidence for confirming the system’s reliability.

Other authors use the Fractional Fourier Transform (FrFT) for watermarking since there is a strong dependency between the orders and the resulting coefficient set of the FrFT, a dependency that seems random. The algorithm proposed in [19] uses the FrFT coefficients as the embedding domain. The authors report good results; however, they present just a case of study. A similar approach is presented in [20]. This approach also uses the fractional orders as the secret keys. The watermark is detected using standard cross-correlation. The authors claim that the system is robust toward JPEG compression, noise addition, and image manipulation operations such as median filtering, Gaussian smoothing, and sharpening filtering. Another work that uses the FrFT is [21]; its authors affirm that their proposal is robust to geometrical transform, filtering, and histogram stretching; however, they carried out too few experiments. In [22], the authors present an approach based on the FrFT with a random modification to the phase. The resulting system is more similar to a digital signature based system than to a typical watermarking system. This system is robust against cropping, salt and pepper noise addition, uniform noise addition, Gaussian noise addition, noise addition in both the amplitude and the phase, JPEG compression, and histogram equalization operations. Another idea presented in [23] is to generate a watermark in the FrFT domain and embed it into an image also in the FrFT domain using the additive rule. The authors used the cross-correlation for detecting the watermark. This scheme is robust toward occlusion attack, which is the only attack reported by the authors.

The Random Fractional Fourier Transform (RFrFT) is a variation of the FrFT; it has the same properties of FrFT but has the advantage that the spectrum is random and exhibits a high embedding capacity and robustness for watermarking applications. An RFrFT application to watermarking is presented in [24]. This system computes the RFrFT with a given random phase; then, it divides the transformed image into blocks and computes their fractal dimension; next, it selects a set of those blocks and uses the highest amplitude in each block for watermark embedding using Amplitude Shift Keying (ASK). The watermark extraction is accomplished by reversing previous steps. The system computes the Mean Square Error to measure the robustness using both the extracted and the real watermark. They tested their system by performing three attacks: noise addition, cropping, and JPEG compression.

Another fractional calculus based transform, the Discrete Fractional Random Transform (DFRNT), was used in [25]. This work is similar to [24]; first, the system computes the DFRNT; then, it divides the signal into blocks and selects a set of blocks randomly; next, it selects the highest amplitudes for watermark embedding using Phase Shift Keying (PSK). The authors report that their proposal is robust against Gaussian noise addition, cropping, and low pass filtering; however, they present too few tests.

One more fractional based transform is the Fractional Dual-Tree Complex Wavelet Transform (FrDT-WT); the FrDT-WT is used to find the wavelet transform in the Fourier domain resulting in a mathematical description of the multiresolution properties. The work presented in [26] and exploits that the randomness of the FrDT-WT coefficients depends on the fractional order, also using a biometric pattern to further enhance the security. The main idea is to build two biometric images; then, use the SURF algorithm to compute the robust matching point vectors; next, use these vectors to compute the keys for building a chaotic map. The watermark extraction uses both the original and the watermarked images. The authors report that their system is robust. The attacks covered in the test include average filtering, median filtering, Gaussian noise addition, salt and pepper noise addition, JPEG compression, SPIHT compression, row-column deletion, resizing, cropping, rotation, histogram equalization, contrast adjustment, and sharpen attacks; however, there were only six images used for the test; furthermore, the reported results correspond to their best case.

Another work is [27] that is almost the same as the system presented in [26]. The main difference between these works is that Ref. [27] uses the Redundant Fractional Wavelet Transform (RFrWT) due to a problem with the discrete FrDT-WT related to the use of decimators.

The authors of [28] present an interesting idea; unlike most watermarking schemes, their system does not embed a watermark into a host image, but they use Visual Cryptography (VC) and a Visual Secret Sharing Scheme. The system constructs two shares that convey a secret message in the following way: the encoder divides the host image into blocks; then, it selects a set of blocks and computes the FrFT using orders α and β; next, it computes the Singular Value Decomposition (SVD) of the transformed blocks and uses the first value of the resulting SVD for computing the master share according to the standard rules of secret sharing schemes. The authors report that their scheme resists various signal processing operations such as JPEG compression, average filtering, median filtering, blurring filtering, sharpening filtering, Gaussian noise addition, contrast adjustment, gamma correction, histogram equalization, resizing, rotation, and geometrical distortion.

All of these works have in common the use of (1) and (2) to embed and detect watermarks; from this perspective, we can say that the overall difference among them is the use of some transform coefficient set for watermarking. In other words, they use already proposed equations, and the novelty of these works rely on the use of a different embedding domain. This leads to incrementing the complexity of the watermarking system and other problems related to the multiple definitions of fractional operators proposed until now.

On the other hand, the authors of [29] analyzed the watermarking systems proposed in [17] through [28] and observed that they use (1) and (2), so they proposed a new improved equation set to substitute (2) and (3). They showed that this modification increases the system’s robustness, so the watermarking system designer might prefer to use fractional equations as a reliable solution for copyright enforcement; however, they limited their study to the case where the watermark is binary, and they added that they would skip the case of Gaussian watermarks since the system based on (2) and (3) was not reliable in this case, so a fair comparison to their proposed equation set was not possible in the context of the experiments carried out to test their proposed scheme.

The authors of [30] explored the case of Gaussian watermarks, and their results suggest that the scheme proposed in [29] reduces the False Positive Percentage; however, they limited the benchmark corpus to 20 images from the standard image set.

The case of the fractional scheme proposed in [30] for the Gaussian watermarks case needs a deeper study; for this reason, we accomplished this study where we explore the behavior of the fractional scheme for detecting Gaussian watermarks; we looked for confirming that the fractional scheme proposed in [29] reduces the false positive percentage of the detector when Gaussian watermarks were embedded, and, by reaching this goal, we confirmed that the fractional scheme is reliable for watermarking applications when Gaussian watermarks are used; thus, the novelty of this paper is to generalize the results presented in [29,30]. The main advantage of the proposed scheme is that it avoids the problems related to the use of fractional transforms found in previous works, keeping the complexity almost the same, however, for detecting Gaussian watermarks.

## 4. Fractional Calculus Approach to Watermark Detection

**Figure 6:**
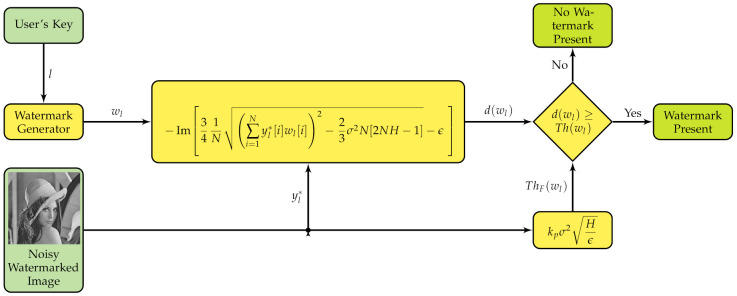
Fractional watermark detecting scheme.

The detection variable derived from FC principles proposed in [29] is:

(5) dF(wl)=−Im341N∑i=1Nyl*[i]wl[i]2−23σ2N[2NH−1]−ϵ,

where Im[·] is the imaginary part operator, and the threshold is:

(6) $$Th_{F}\left(w_{l}\right)=k_{p}\sigma^{2}\sqrt{\frac{H}{\epsilon }},$$

where:

(7) $$\epsilon =\frac{3}{4}\frac{1}{N}\sqrt{\frac{2}{3}\sigma^{2}N\left[2NH-1\right]},$$

with $H=\ln(\sqrt{2\pi \sigma^{2}e})$; $\sigma^{2}$ is the variance of $y_{l}^{*}$. We call (5) and (6) the Fractional equation set. A fractional scheme based on (5) and (6) is shown in Figure 6.

The decision of the system for the fractional equation set is:

(8) $$D_{F}\left(w_{l}\right)=\left\{\begin{array}{cc} 1 & d_{F}\left(w_{l}\right)\geq Th_{F}\left(w_{l}\right)\\ 0 & \mathrm{otherwise} \end{array}\right..$$

**Figure 7:**
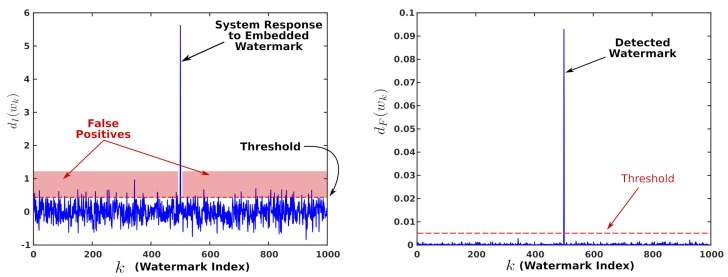
Detection of the watermark. (**left**) using (2) and (3); (**right**) using (5) and (6), and note the lack of False Positives. The cases that fall in the red zone are False Positives.

If we use (5) and (6) for detecting Gaussian watermarks, we get the result shown in Figure 7, which clearly has improved detection characteristics since it has no false positives. We are looking for confirming that the scheme in Figure 6 is more reliable than the scheme in Figure 5. This follows the strategy stated early in this paper about reusing the algorithm in Figure 5 by replacing detection equations with fractional calculus based equations resulting in a possibly improved algorithm and then verifying the effectiveness of this strategy. For this reason, there is a lack of comparison to related works as a means of control to the experiments. In other words, a fair experiment in the purpose’s context of this paper is to compare the original algorithm versus the same algorithm with fractional equations and assess its improvement. Thus, the only control needed is the original algorithm and, as a result, the outcomes of the experiments are reliable.

## 5. Materials and Methods

To carry out experiments, we used 10,000 images of the BOWS database as the set X; each image of this set is grayscale with size 512×512 pixels and their luminance values are in the range [0,255].

We used the embedding scheme shown in Figure 3 for watermarking each image in the set X. In addition, the embedding gain was fixed for all cases to the value g=5; this setting leads to a Peak Signal-to-Noise Ratio (PSNR) mean of 34.21 dB for the entire set giving a fair balance between robustness and imperceptibility of the watermark. The embedded watermark $w_{l}$ was selected at random from the watermark set W for each image; each watermark in the set was equally probable.

The goal of the tests was to assess the capacity of watermarking schemes shown in Figure 5 and Figure 6 to reduce the false positives by computing the FPP and the ROC curve.

**Figure 8:**
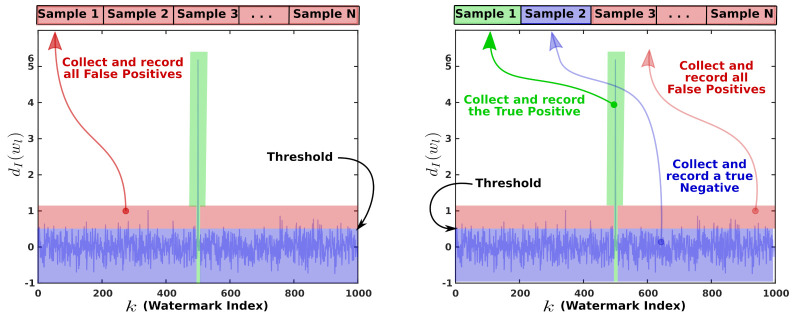
Collecting data. (**left**), all the system’s responses that cross the threshold when no watermark was embedded are collected since these are False Positives. These data fall in the red zone. (**right**) We collected the true positive (data in the green zone), a single true negative (data in the blue zone), and all false positives for each image in the set Y (data in the red zone).

For the first test, we computed the FP of each image. To achieve this, each image in the set X was watermarked; then, the system tried to detect all watermarks in W within a single image; next, all FP were identified and counted using (4) and (8). The false positives computing process is summarized in Procedure 1, and it is described in Figure 8 (left). False positives gave us an insight about the reliability of both the integer and the fractional schemes.

For the sake of simplicity, and without loss of generality, we indicated $D(w_{l})$ instead of $D_{I}\left(w_{l}\right)$ or $D_{F}\left(w_{l}\right)$ in all procedures since the same steps were followed for both schemes.

We selected the Receiver Operating Characteristic (ROC) since it is regarded as an objective measure to evaluate performance of a decision technique. Thus, as a second test, we computed the ROC as follows: each image in the set X was watermarked using watermark $w_{l}$; then, we computed $d_{I}\left(w_{l}\right)$, $d_{F}\left(w_{l}\right)$, $D_{I}\left(w_{l}\right)$, $D_{F}\left(w_{l}\right)$. Those values and the corresponding ground truth values of $D_{I}\left(w_{l}\right)$, $D_{F}\left(w_{l}\right)$ were recorded. The data were used to derive a Generalized Lineal Model for estimating the ROC for both the integer and the fractional schemes. Data collecting steps were summarized in Procedure 2 and further explained in Figure 8 (right). The ROC curves were used to evaluate the integer and the fractional to clarify which of them is more reliable.

With a last test, we examined the robustness of the fractional scheme. To achieve this goal, each image in the set X was watermarked to get the set of watermarked images Y, and then an attack was carried out on the watermarked images; next, we added up the cases where the embedded watermark was detected to compute the percentage of detected watermark cases. The process of computing the detection rate is summarized in Procedure 3. The percentage of detected watermarks after the watermarked image was attacked suggested the target applications of the fractional scheme based on its robustness.

**Procedure 1** Procedure to record measures for False positives.**Require:** Image set X, Watermark set W. 1: Open log file for writing. 2: **for** Each image $x_{k}\in X$
**do** 3: Select randomly a watermark $w_{l}$ from the watermark set W. 4: Partition set W into two subsets $W_{e}$ and $W_{n}$ that hold $W_{e}\cap W_{n}=\emptyset$ and $W_{e}\cup W_{n}=W$, $W_{e}=\left\{w_{l}\right\}$. 5: Embed the watermark $w_{l}$ into Image $x_{k}$ 6: **for** Each watermark $w_{m}$ in $W_{n}$
**do** 7:  **for** Each watermarking scheme **do** 8:   Compute $R(w_{m})$ 9:   **if**
$R(w_{m})$==1 **then**10:    FP=FP+1
11:   **end if**
12.  **end for**
13: **end for**
14: Record FP a of a current image in log file.15: **end for**
16: **return** Log file.


**Procedure 2** Procedure to record measures for getting the ROC curve.**Require:** Image set X, Watermark set W. 1: Open log file for writing. 2. **for** Each image $x_{k}\in X$
**do** 3: Select a random watermark $w_{l}$ from the watermark set W. 4: Embed the watermark $w_{l}$ into Image $x_{k}$. 5: Compute $d(w_{l})$ and $D(w_{l})$. 6: Record $d(w_{l})$, $D(w_{l})$, and ground truth values in log file. 7: Get the set of indexes of true negatives, these indexes form a set T.
 8: Draw at random an index *m* from set T. 9: Record $d(w_{m})$, $R(w_{m})$, and ground truth values.10: Get the set of indexes false positives, these indexes form a set P.11: **for** Each index *k* in P
**do**12:  Record the values of $d(w_{m})$, $R(w_{m})$, and ground truth values in log file (Record all the False Positives).13: **end for**
14: **end for**
15: Close log file.16: **return** Log file.


**Procedure 3** Procedure to record Detection Rate.**Require:** Image set X, Watermark set W. 1: Open log file for writing. 2: **for** Each image $x_{k}\in X$
**do** 3: Select randomly a watermark $w_{l}$ from the watermark set W. 4: Embed the watermark $w_{l}$ into Image $x_{k}$ 5: Perform an attack on the watermarked image. 6: Compute $D(w_{l})$ using the attacked image. 7: **if**
$D(w_{l})$==1 **then** 8:  D=D+1
 9: **end if**
10: **end for**
11: Compute detection rate ($D_{r}=\frac{D}{10,000}$)12: Record $D_{r}$ in log file.13: **return** Log file.


## 6. Experimental Results

**Figure 9:**
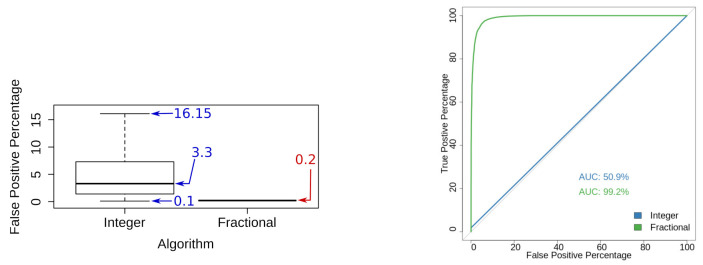
Statistical assessment of discrimination characteristics. (**left**) value ranges of false positives for both the Integer and Fractional watermarking schemes. No outliers are drawn for the sake of clearness; (**right**) comparison of the ROC curves of the integer and fractional schemes.

As a first test, we computed the false positive percentages for both the integer and the fractional schemes and build a boxplot. Figure 9 (left) shows that the false positives for the integer scheme span from 0.1% to 16.15%; in contrast, the fractional scheme has very low percentages of false positives and the range of values concentrates around 0.2%.

In our second test, we evaluated the quality of both schemes; we used the data we collected to draw the ROC curve shown in Figure 9 (right). As a result of this test, we found that the ROC of the integer scheme has an Area Under the Curve (AUC) of 50.9%, whereas the fractional scheme obtained an AUC of 99.2% for the same test.

The last test consisted of examining the successful detection rate after attacks; this was accomplished by attacking each watermarked image in the set Y. The attacks performed were: average filtering, median filtering, Gaussian noise addition, speckle noise addition, salt and pepper noise addition, JPEG compression, cropping, removing random rows and columns, substituting random rows and columns, and scaling. The corresponding figures are in Appendix A for the sake of readability of this section.

**Figure A1:**
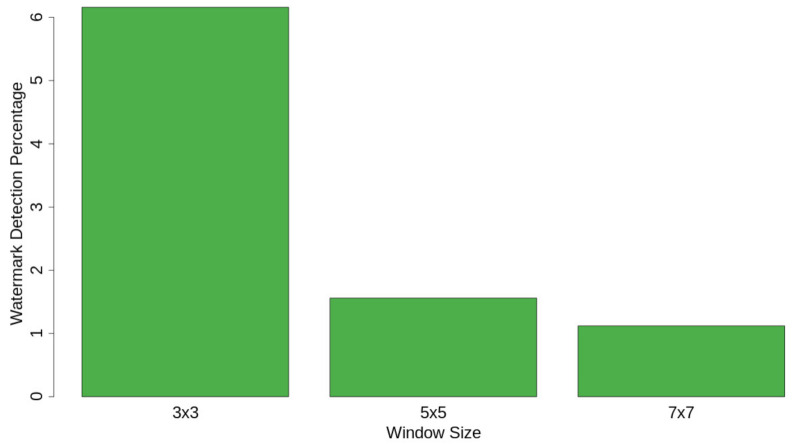
Percentage of successful watermark detection after an average filter attack for various window sizes.

A bar plot of the percentage of detected watermarks after the image set Y filtered using an average filter is shown in Figure A1. The attack was repeated for window sizes of 3×3, 5×5, and 7×7. The resulting bar plot shows that the percentage of successful watermark detection after the attack is about 6% and became lower as the window size increases.

**Figure A2:**
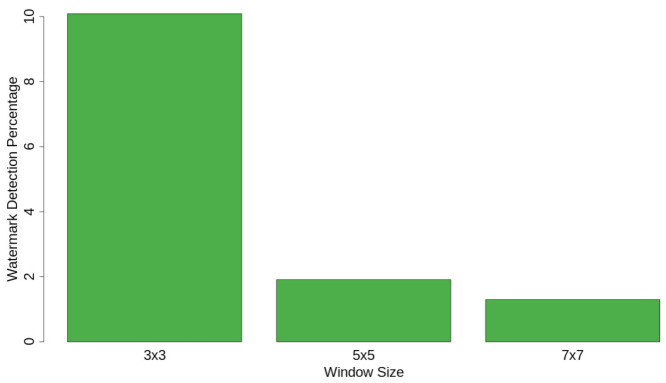
Percentage of successful watermark detection after a median filter attack for various window sizes.

We performed a similar attack; this time, a median filter was used to filter the set Y. Results shown in Figure A2 reveal a similarity to the results reached for the average filtering attack; this time, detection percentages are lower than 10%.

**Figure 10:**
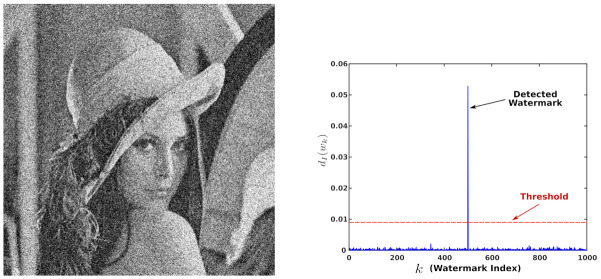
Detection of the watermark. (**left**) noisy watermarked imaged; noise variance was 0.05. (**right**) the corresponding evaluation of (5).

**Figure A3:**
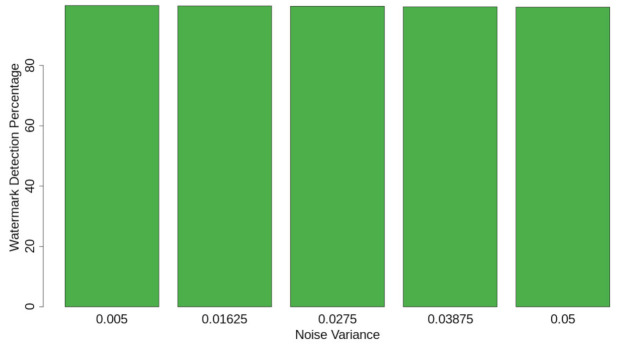
Percentage of successful watermark detection when Gaussian noise is added to the watermarked image. The mean of the noise was zero and the horizontal axis corresponds to the variance of the noise.

The next test is comprised of adding Gaussian noise to each watermarked image in the set Y and then we tried to detect the watermark. We constructed a bar plot showing the percentages of detected watermark for various noise variances. Figure A3 shows that the scheme is robust to Gaussian noise. This figure might look suspicious because it looks atypical; thus, to discard that the Gaussian noise triggers false positives and this causes a high detection rate, we inspected some cases and present an example in Figure 10.

**Figure A4:**
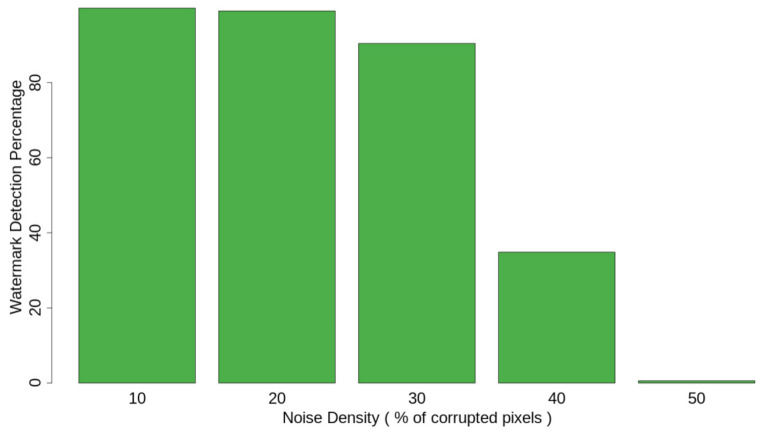
Percentage of successful watermark detection when salt and pepper noise is added to the watermarked image. Horizontal axis show the noise density.

The following test consists of adding salt and pepper noise and then detecting the watermark. Results depicted in Figure A4 show that the fractional scheme is robust up to noise densities of 20%, the detection rate drops for noise densities higher than 20%.

**Figure A5:**
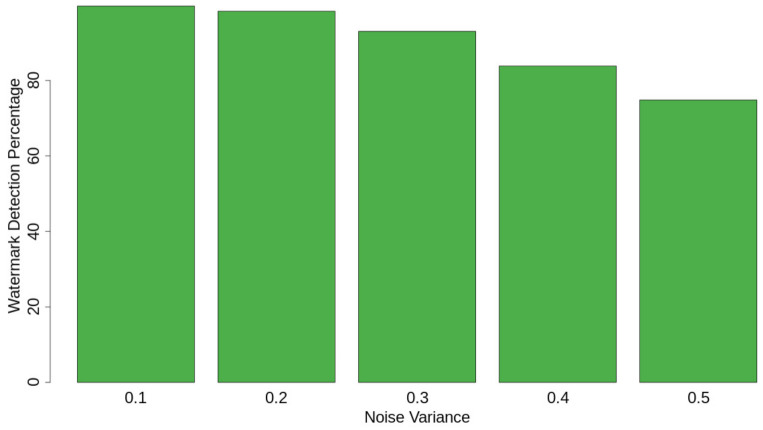
Percentage of successful watermark detection when speckle noise is added to the watermarked image. Horizontal axis show the noise variance.

We carried out the next test by adding speckle noise before trying to detect the watermark. Results in Figure A5 show that the fractional scheme is robust up to noise variances of 0.2. After this limit, the detection rate drops.

**Figure A6:**
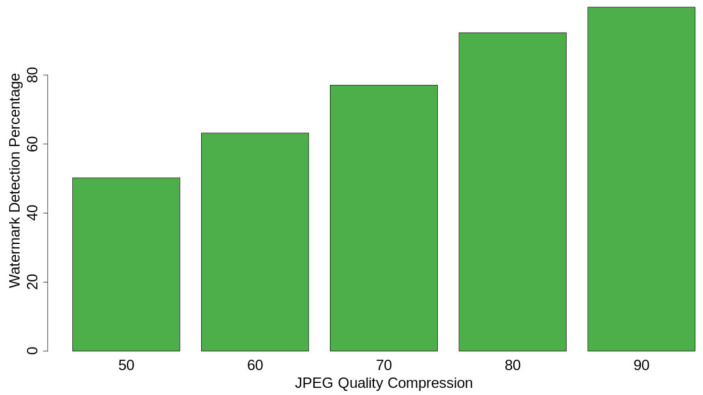
Percentage of successful watermark detection after the watermarked image was compressed using the JPEG standard. The horizontal axis corresponds to the JPEG compression quality factor.

A very common scenario is to compress images using the JPEG standard, so the next test comprised watermarking the image and compressing the image with the JPEG standard, and then detecting the watermark. Figure A6 shows that the fractional scheme is robust to JPEG compression up to a quality factor of 90% and then the detection percentage starts to decline.

**Figure A7:**
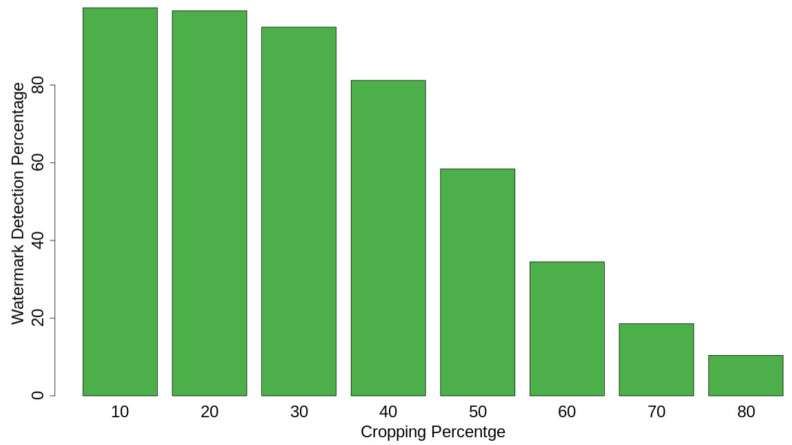
Percentage of successful watermark detection after cropping. This figure spans various cropping percentages.

Another common signal processing operation is the cropping attack. Figure A7 shows results when the watermarked image is cropped. This figure shows that the fractional scheme is robust up to 20% of cropped pixels.

**Figure A8:**
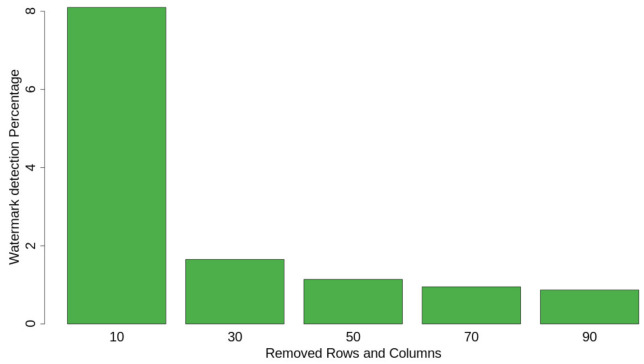
Percentage of successful watermark detection after a number of rows and columns were removed from the watermarked image. The horizontal axis shows the number of rows and columns removed.

The next test selects *t* rows and *t* columns at random, and then removes these rows and columns from the watermarked image; the resulting image is smaller than the original watermarked image, so the image is then scaled to match the size of the original watermarked image. The watermark was then detected, and the results are shown in Figure A8. Results show that the fractional scheme is not robust since it exhibits a detection rate around 8% for removing 10 rows and columns.

**Figure A9:**
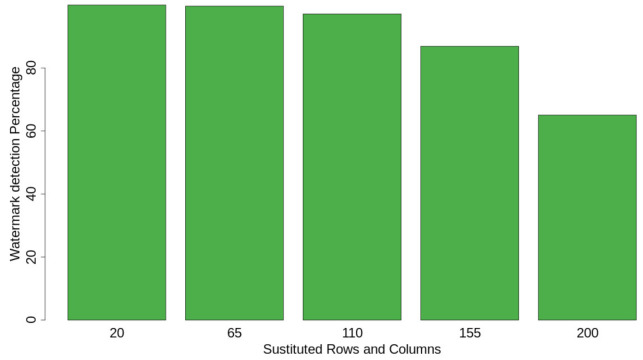
Percentage of successful watermark detection after a number of rows and columns of the watermarked image were substituted with another row or column.The horizontal axis shows the number of rows and columns replaced.

Another test, similar to the previous one, selects *t* rows and *t* columns at random, and then substitutes the selected rows and columns with the adjacent row or column of the same image. The watermark was detected, and results are shown in Figure A9. Results show that the fractional scheme is robust up to substituting 100 rows and columns.

**Figure A10:**
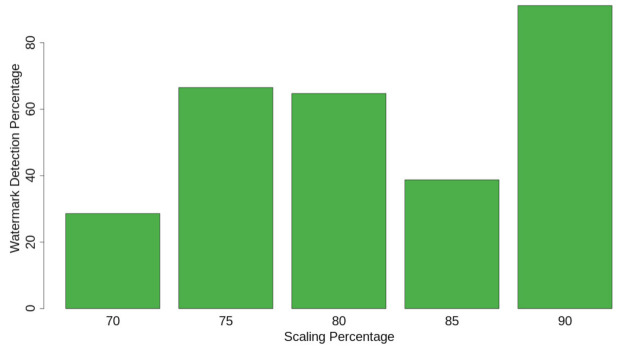
Percentage of successful watermark detection when the watermarked image is scaled. The horizontal axis shows the scaling factor.

Finally, we carried out a scaling attack; the watermarked image was scaled to make it smaller and then the image was restored back to its original size. The results are shown in Figure A10; this figure shows that the fractional scheme is robust up to 90%. In other words, we shrank the image to 90% of its original size and then restored to the original size before we tried to detect the watermark.

## 7. Conclusions

In this study, we compared the FPP of the original watermarking scheme versus the corresponding version with detection equations derived from fractional calculus; evaluated the quality of both schemes as a watermark detector by comparing their ROC curves, and examined the successful detection rate after attacking the watermarked images to define robustness of the system. We performed several tests that allowed us to conclude the following facts:

The False Positives percentage is much lower for the fractional scheme than the corresponding percentages of the integer scheme. According to Figure 9 (left), the FPP spans from 0.2% to 16.2% for the integer scheme whilst the FPP concentrates around 0.2% for the fractional scheme. This means it is more likely to get a 0.2% FPP when using a fractional scheme and also the FP rate will be lower for this fractional scheme than the corresponding results for an integer scheme.

Results show that the fractional scheme is a reliable method for detecting Gaussian watermarks according to Figure 9 (right); the fractional scheme has a significant advantage compared to the integer scheme since the AUC is higher for the fractional case (AUC=99.20% versus AUC=50.90%); this means that the fractional scheme has higher discriminative power compared to the integer scheme.

In addition, the experimental results in Figure A1–Figure A10 show that this system is fragile to all attacks presented in Section 6, except for the case of the Gaussian noise addition attack, this is because the noise is added in the same manner as the watermarks are; thus, the systems treats Gaussian noise as a watermark. The target applications of such a scheme include cases where the watermarks should not survive attacks; an example of practical application of a fractional scheme is for authenticating information.

Since the target application might be authenticating information, it will be convenient to propose another value of $k_{p}$; this value should be higher than that used in this study since this will help to reduce the detection rate after attacks. Additional usage scenarios include: The system designer wants to enhance the discriminative power of a system already proposed, the watermark is some information that closely holds the Gaussian distribution, and the complexity of the watermarking system has to be low.

The results provide designers of watermarking systems with an alternative to take advantage of Gaussian watermarks when appropriate to meet their design goals. Thus, the proposed strategy is an alternative to reuse previously proposed schemes by using fractional calculus based equations.

The results obtained in this study complement the study in [29] since the case of Gaussian watermarks was left unexplored, so this paper provides the designer of watermark systems with a more logical insight of the potential and practical applications of a fractional watermark detector.

The characteristics to discriminate between patterns with Gaussian statistical distribution suggest that the fractional equations might be used in pattern recognition applications where samples have a Gaussian distribution.

## Data Availability

Not applicable.

## Appendix A

This appendix has complementary experimental data that the reader might need to check out closer.
